# Polarity- and Pressure-Induced Emission from a Benzophenone-Based Luminophore

**DOI:** 10.3390/molecules27248748

**Published:** 2022-12-09

**Authors:** Qintao Hu, Pu Zhang, Yunpeng Zhang, Jingwei Sun

**Affiliations:** Department of Materials Chemistry, Huzhou University, Xueshi Road No.1, Huzhou 313000, China

**Keywords:** solvatochromism, mechanochromism, molecular conformation

## Abstract

Since strong polarity usually causes emission quenching, materials with polarity-induced emission (PIE) are rarely reported despite their important applications in polar environments. Herein, an *N*-phenylcarbazole-substituted benzophenone derivative (BP-3-Cz) with a twisted electron donor–acceptor (D–A) structure is synthesized. The incorporation of heteroatoms into the twisted π-conjugated D–A backbone simultaneously endows BP-3-Cz with obvious polarity- and pressure-induced emission. Spectral analysis, X-ray diffraction, differential scanning calorimetry, and quantum chemical calculation results confirm that BP-3-Cz has special optical features related to the molecular conformation change and excited state turning to planarized intramolecular charge transfer with an increase in polarity or applied pressure. These findings contribute to the understanding of the PIE mechanism and the design of new PIE materials.

## 1. Introduction

Multiple stimulus-induced emission materials, which are nonemissive but become highly emissive after contacting multiple stimuli or undergoing different treatments, are attracting considerable interest as promising candidates for use in multifunctional luminogens, sensors, security inks, optical memories, and displays [1,2,3,4]. The stimulus responses for fluorescence turn-on include many mechanisms, such as aggregation-, pressure-, polarity-, chemically, and thermally induced emission [5,6,7,8]. Among them, aggregation-induced emission (AIE) is being comprehensively investigated in both the fundamental research and industrial fields, as the processes for and practical difficulties in accessing them by conventional ACQphores can now be readily solved by AIEgens [9]. In addition, pressure-induced emission materials, which belong to one type of mechanochromic materials that exhibit fluorescence upon the application of pressure, have also been studied recently owing to their high-contrast luminescence switching [10,11]. By contrast, polarity-induced emission (PIE), where fluorophores are quenched in nonpolar and low-polarity solvents but show strong emission at high polarities, is rarely reported because strong polarity usually causes emission quenching [12]. Materials that exhibit aggregation-, pressure- and polarity-induced emission simultaneously are even rarer.

Normally, materials with electron donor–acceptor (D–A) structures, which have intramolecular charge transfer (ICT) properties, are strongly sensitive to surrounding polarity [13,14]. However, traditional D–A molecules are weakly emissive or nonemissive in high-polarity solvents but exhibit strong luminescence in nonpolar solvents due to twisted ICT (TICT) [15,16,17]. The severe charge separation caused by a twisted molecular conformation is detrimental to radiative decay and thus leads to weak fluorescence in high-polarity solvents [18]. Considering the importance of PIE materials for applications in polar environments, their development and studies on their structure and mechanism, albeit challenging, are urgently needed.

**Scheme 1:**
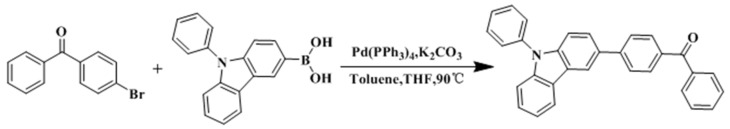
Synthesis route of BP-3-Cz.

Herein, we synthesized an N-phenylcarbazole-substituted benzophenone derivative (BP-3-Cz) with a twisted D–A structure, as shown in Scheme 1. BP-3-Cz has been reported to show an efficient AIE property and has been successfully applied as an emissive layer in deep-blue OLEDs. The nondoped OLED exhibited an EQE_max_ and ultrapure CIE coordinates of 3.7% and (0.15, 0.15), respectively, with very small efficiency roll-offs [19]. In this present work, BP-3-Cz is found to exhibit PIE and pressure-induced emission simultaneously. It has a faint fluorescence in n-hexane (Hex), with a photoluminescent quantum yield (PLQY) of only 0.3%. BP-3-Cz exhibits especially strong fluorescence (PLQYs exceeding 90%) in high-polarity N,N-dimethylformamide (DMF) and acetonitrile (ACN). An especially weak emission (PLQY 0.4%) is observed from BP-3-Cz in its crystalline state. In contrast, the PLQY is improved by more than 36 times after grinding. Such multiple stimulus-induced emission properties of BP-3-Cz have not yet been reported. Spectral analysis, X-ray diffraction (XRD), differential scanning calorimetry (DSC), and quantum chemical calculations were performed to explore this unusual phenomenon. This work contributes to the understanding of the PIE mechanism and the design of new PIE materials.

## 2. Results and Discussion

### 2.1. Polarity-Induced Emission

BP-3-Cz was synthesized via the Suzuki reaction outlined in Scheme 1. The detailed synthesis route and structural characterization, including ^1^H NMR, ^13^C NMR, MS, and FTIR data are shown in the Materials and Methods section. 

**Figure 1:**
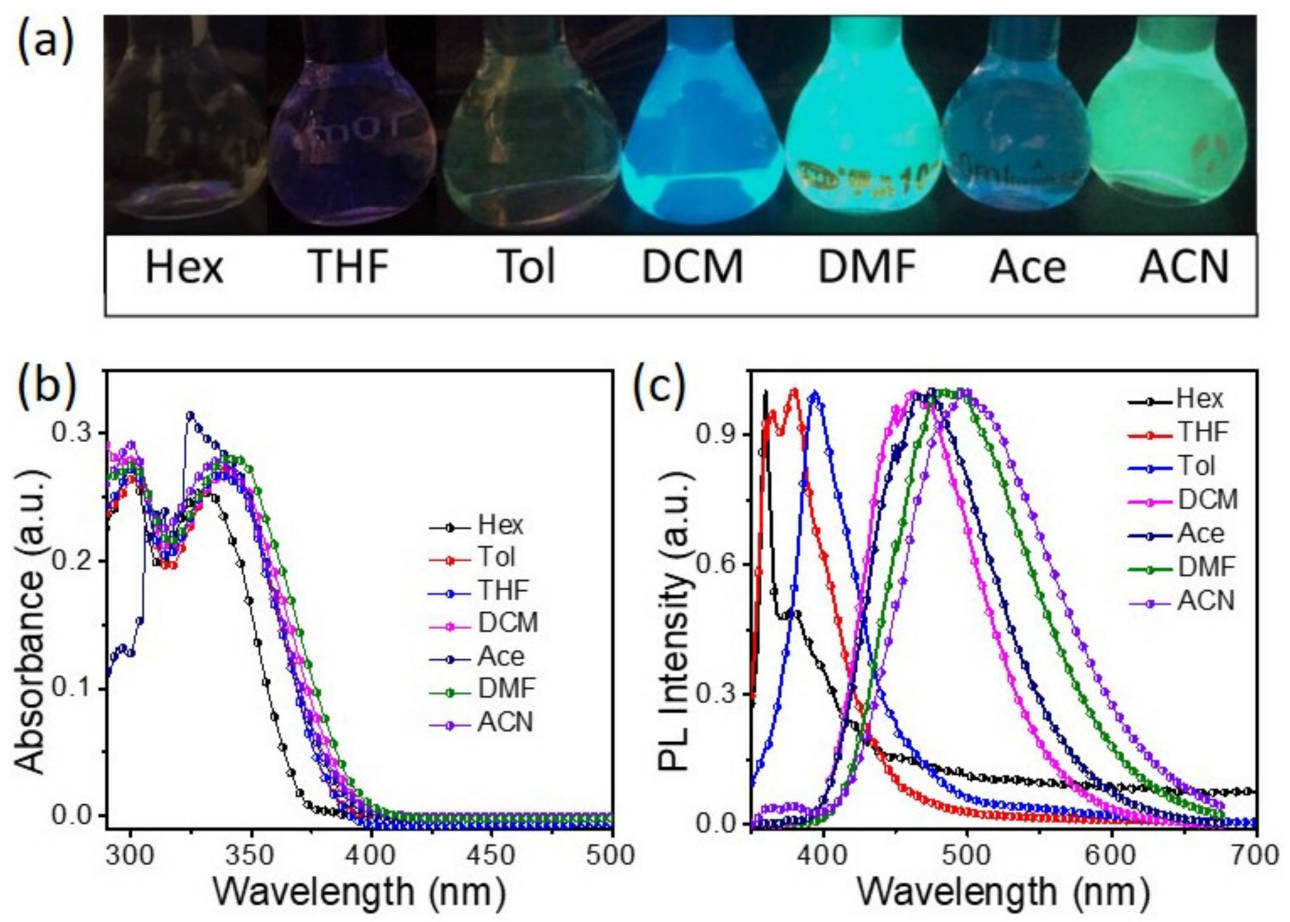
(**a**) Fluorescence images and (**b**) absorption and (**c**) PL spectra of BP-3-Cz in solutions with different polarities.

The basic optical properties of BP-3-Cz were investigated in solvents of varying polarities. Generally, strong polarity often causes emission quenching. However, BP-3-Cz exhibits an opposite phenomenon. As shown in Figure 1a, no fluorescence is observed from BP-3-Cz in Hex, and weak deep-blue and cyan-blue fluorescence shows in tetrahydrofuran (THF) and toluene (Tol), respectively. Notably, with a continuous increase in polarity, BP-3-Cz exhibits particularly strong fluorescence in dichloromethane (DCM), DMF, acetone (Ace), and ACN. The absorption and photoluminescence (PL) spectra of BP-3-Cz are shown in Figure 1b,c, respectively. In Hex, BP-3-Cz shows two main absorption bands at about 300 and 330 nm, which originate from the π-π* and ICT transition, respectively [20]. Meanwhile, in Tol and polar solvents, BP-3-Cz exhibits an absorption band around 300 nm that is almost identical to that in Hex and largely redshifted ICT bands around 340 nm. These ICT bands change a little with a continuous rise in polarity. In comparison, a remarkably redshifted PL peak from 370 to 500 nm is observed in BP-3-Cz when the solvent changes from Hex to ACN. In Hex and THF, the compound shows narrow emission peaks with vibrational structures at short wavelengths that correspond to localized emission (LE) [21]. The larger redshift of the PL peak in Tol compared with that in THF is ascribed to the conjugation effect with Tol. With a further increase in solvent polarity, the emission band gradually broadens along with the disappearance of the vibronic resolution. Meanwhile, BP-3-Cz shows a substantial redshift of more than 100 nm and finally moves to 500 nm in ACN. This continuous shift of the PL peak is attributed to ICT emission. These spectral results, similar to those of most D–A molecules, indicate a clear ICT in BP-3-Cz, which is highly significant in polar environments [22]. The charge transfers from the electron-rich carbazole to the electron-withdrawing benzophenone group. However, distinctly, BP-3-Cz exhibits enhanced emission in high-polarity solvents. 

**Figure 2:**
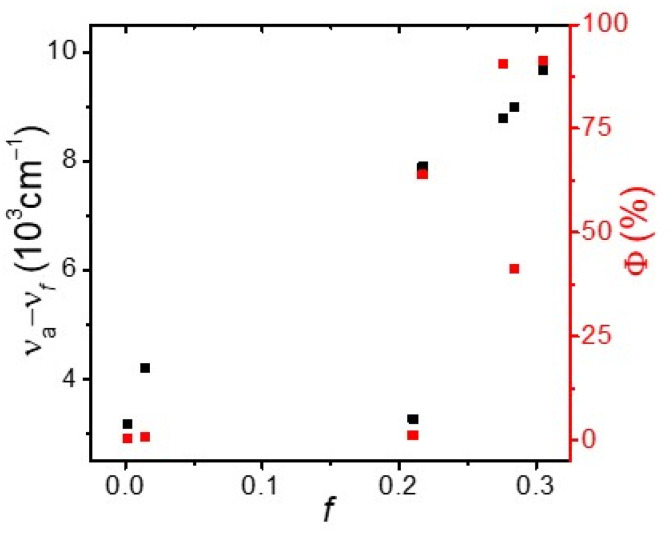
Stokes shifts (black point) and PLQYs (red point) of BP-3-Cz in different solvents.

Detailed photophysical data of BP-3-Cz in the various solvents are listed in Table 1 for an understanding of its unusual fluorescence properties. We further studied the relationship of the Stokes shifts (*ν_a_* − *ν_f_*) and PLQYs with the orientation polarizability (*f*), as shown in Figure 2. *f* is defined as:

(1) $$f=\frac{\varepsilon -1}{2\varepsilon +1}-\frac{n^{2}-1}{2n^{2}+1},$$

where ε is the static dielectric constant and n is the optical refractive index of the solvent [23]. In nonpolar and low-polarity solvents (*f* < 0.217), both the Stokes shift and PLQY are very low, which may indicate the self-absorption of BP-3-Cz. When *f* > 0.210, the Stokes shift displays an almost linear relation with *f*, which has a relatively large slope, further implying the presence of ICT in BP-3-Cz [24]. Moreover, the PLQY abruptly increases to more than 60% in DCM, and even exceeds 90% in DMF and ACN. The relatively low PLQY in Ace is an exception; the absorption of Ace is in the range of 270–330 nm, which partially coincides with that of BP-3-Cz. Nonetheless, an evident PIE is confirmed in BP-3-Cz due to the transition from LE to ICT emission.

Up to now, PIE, where fluorophores are quenched in nonpolar solvents but show remarkable emission in high-polarity solvents, has rarely been reported. This phenomenon may be due to two reasons. For some heteroatom-containing π-conjugated molecules, the coupling of the close-lying dark state (n, π*) and bright state (π, π*) prompts a fast internal conversion (IC) process, leading to weak emission in nonpolar solvents. By contrast, in high-polarity solvents, the difference between the two energy levels is enlarged, so the IC process is suppressed and the emission is released [18]. The other reason is planarized ICT (PICT), where a twisted D–A molecule tends to flatten in a high-polarity solvent to extend the conjugation and increase the orbital overlap in the excited state, thus promoting radiative transition [25]. In BP-3-Cz, the PIE more likely originates from the synergistic effect of both mechanisms. In nonpolar solvents, weak emission may result from a fast IC process due to the close energy levels of (n, π*) and (π, π*). With an increase in solvent polarity, the excited state switches from LE to lower-lying ICT, so the proximity effect of the dark state (n, π*) is suppressed and bright ICT emission is displayed. To verify this supposition, the influence of the conformational planarization on the ICT excited state and photophysical properties of BP-3-Cz is further confirmed and discussed below. 

### 2.2. Pressure-Induced Emission

**Figure 3:**
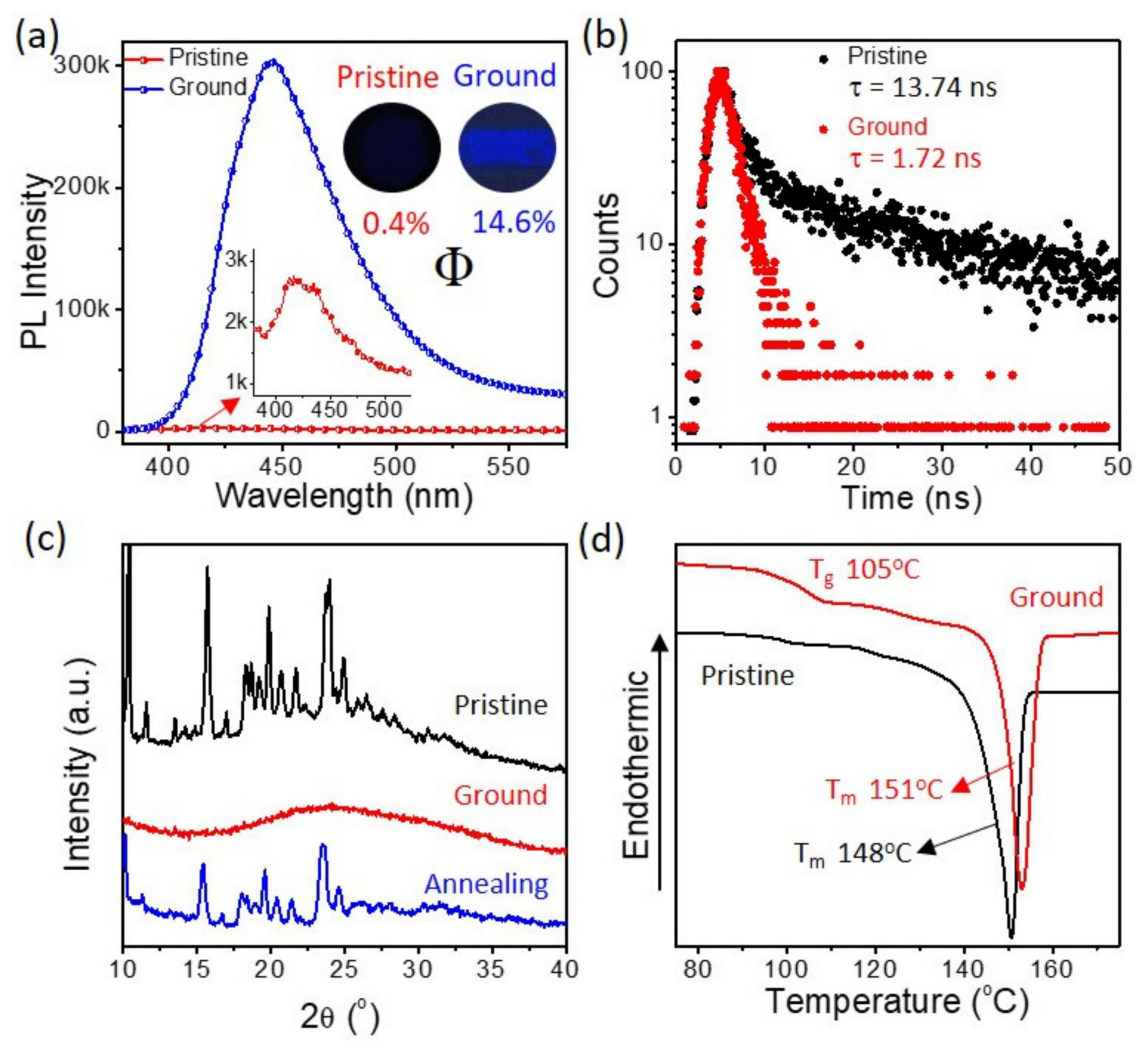
(**a**) PL, (**b**) TRPL, (**c**) XRD, and (**d**) DSC curves of BP-3-Cz in different solid states. The inset of (**a**) shows the corresponding Φ and fluorescence photos.

Although BP-3-Cz shows AIE properties, no fluorescence is observed from its crystalline solid (Figure 3). However, pressure-induced fluorescence emission, which is a kind of mechanochromism, is seen in BP-3-Cz. The pristine powder of BP-3-Cz exhibits a weak PL peak around 425 nm, with a particularly low PLQY of 0.4%, while after the grinding, the PL peak remarkably enhances and shifts to nearly 450 nm, with a high PLQY of 14.6%. Thus, a bright blue luminescence is evident after grinding. The quenched state can be recovered through annealing at 100 °C, indicating the reversible mechanochromism of BP-3-Cz. Figure 3b shows the time-resolved PL (TRPL) curves of the pristine and ground powders. The pristine powder exhibits a double-exponential decay of fluorescence, with lifetimes (τ) of 1.40 (19.35%) and 16.70 ns (80.65%), respectively. While the ground powder exhibits a single exponential decay of fluorescence, with a τ of 1.72 ns. The relatively longer τ of BP-3-Cz in the pristine state might be ascribed to the forbidden electronic transition in the highly twisted conformation of the crystalline lattice, according to the following results.

Powder XRD and DSC were conducted to investigate the structural variation of the samples after grinding. The XRD patterns of the pristine and annealed BP-3-Cz exhibits multiple sharp diffraction peaks in the same positions, revealing that BP-3-Cz adopts a consistent ordered molecular packing structure in both states (Figure 3c). However, the sharp diffraction peaks totally disappear after grinding, indicating that the grinding treatment destroys the ordered packing of the molecules and produces an amorphous phase. The DSC curves of BP-3-Cz before and after grinding are compared in Figure 3d. The melting point of pristine BP-3-Cz is 148 °C, while after the grinding, glass transition occurs at 105 °C, which is similar to the annealing temperature. Furthermore, the melting point noticeably increases to 151 °C. These results reveal that the molecules have much stronger interactions in the ground powder, despite the amorphous structure. Conformational planarization and tight molecular stacking are thought to be induced by the grinding treatment [25]. 

The BP-3-Cz molecule may take a more twisted conformation in the crystalline state, which is favorable for such propeller-shaped flexible D–A molecules [26]. However, the highly twisted conformation in BP-3-Cz leads to suppressed fluorescence emission due to the close-lying S_2_ (n-π*) and S_1_ energies and forbidden electronic transition of S_1_, according to the following theoretical calculation results. Thus, especially long τ and weak fluorescence is observed from its crystalline solid. Grinding disturbs the non-covalent interactions in the crystal and leads to compact molecular stacking, which results in more planarized conformation of BP-3-Cz. Consequently, strong fluorescence is observed in ground powder because the ICT transition is released as a result of the increased orbital overlap between the donor and acceptor [26].

### 2.3. Theoretical Calculation

**Figure 4:**
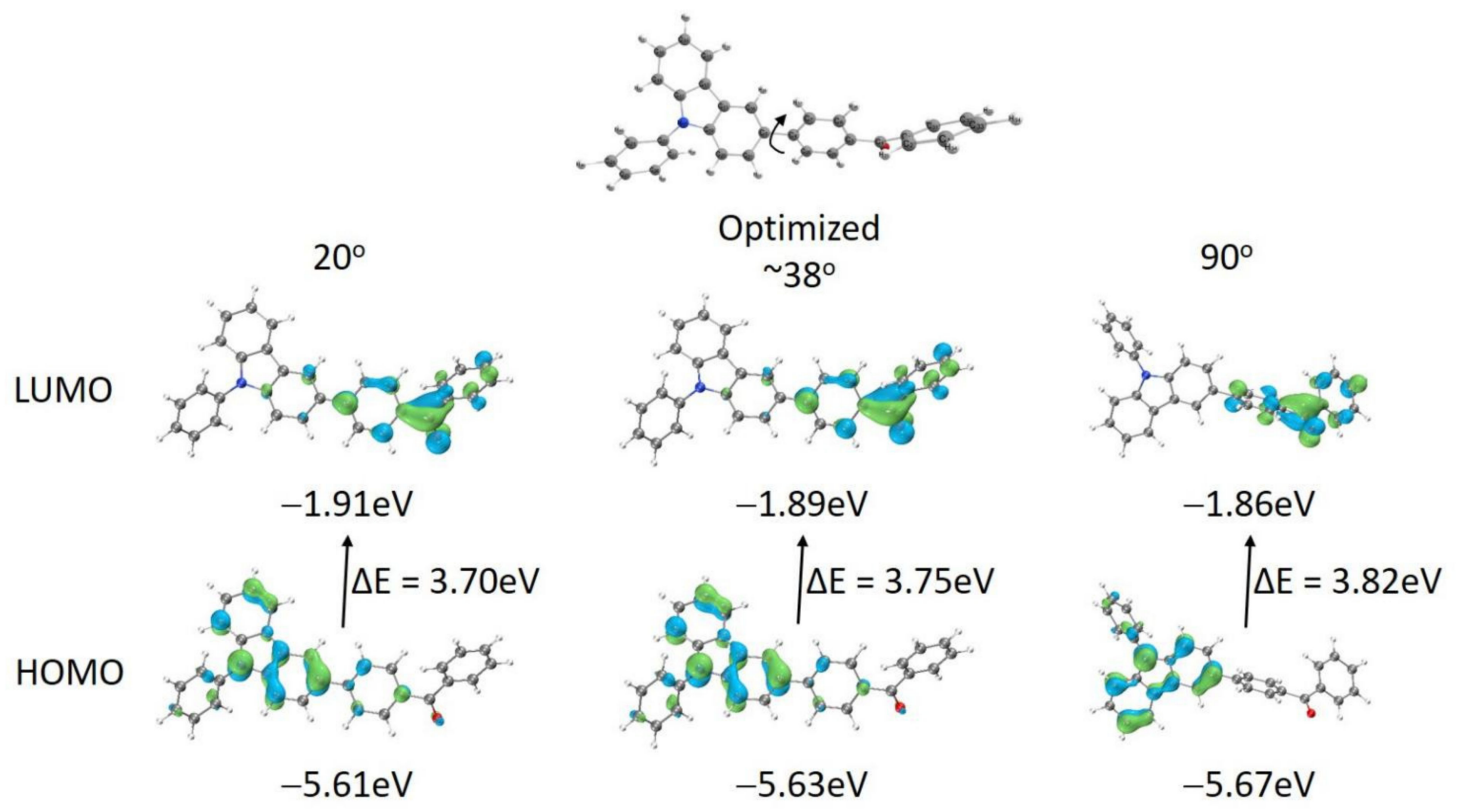
Frontier molecular orbitals (HOMO and LUMO) in the ground state of BP-3-Cz with different twist angles between the carbazole plane and benzophenone unit.

The optimized geometries and spatial distributions of the HOMO and LUMO in the ground state were studied at the DFT level with the B3LYP functional and 6-31G* basis set to gain insights into the geometric and electronic structures of BP-3-Cz. As shown in Figure 4, twisted angles of ~38° are observed between the carbazole plane and the phenyl in the benzophenone connected to it. In addition, the HOMO is located mainly at the N-phenylcarbazole and partially at the benzophenone, whereas the LUMO is distributed mainly at the benzophenone and partially at the carbazole. Thus, BP-3-Cz in the free state contains a minor portion of LE transition and a major portion of ICT transition from the N-phenylcarbazole to the benzophenone group [27]. The calculated 3.75 eV bandgap is consistent with that obtained from the absorption wavelength. To evaluate the transitions of BP-3-Cz in varied conformations, the torsion angle between N-phenylcarbazole and benzophenone is limited to 20° and 90°, respectively. At the low dihedral angle of 20°, the HOMO is further delocalized on the benzophenone and the LUMO is further delocalized on the carbazole. However, at the 90° dihedral angle the HOMO is evidently concentrated in the *N*-phenylcarbazole, whereas the LUMO is totally located at the benzophenone. Therefore, the HOMO and LUMO are fully separated in space, which induces a pure-ICT transition from the N-phenylcarbazole to the benzophenone moiety. Thus, the molecular conformation, which can be changed by the solvent polarity in solutions and the external pressure in solids, affects the optical transition of BP-3-Cz.

**Figure 5:**
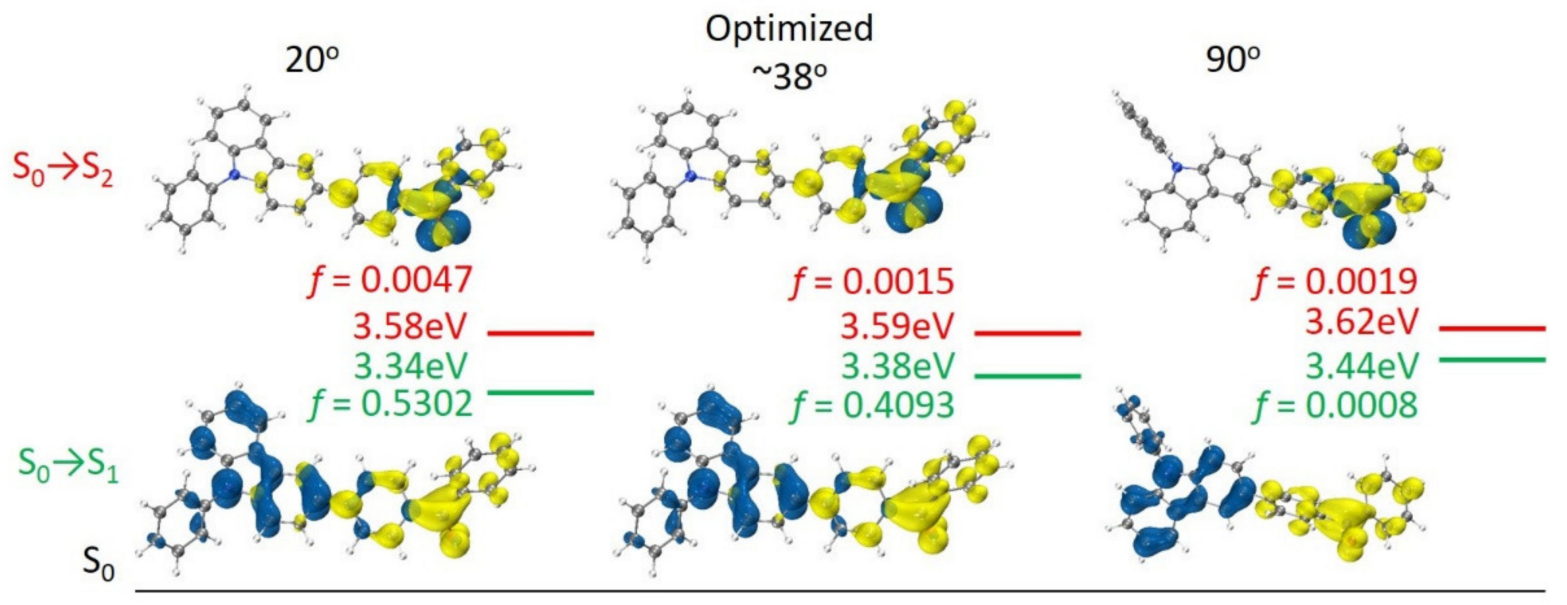
Natural transition orbitals of BP-3-Cz with different twist angles between the carbazole plane and benzophenone unit.

The natural transition orbitals (NTOs) were analyzed using the Multiwfn program to explore the photophysical properties in their excited states [28]. The blue and yellow isosurfaces in Figure 5 represent hole and electron distributions, respectively. *f* represents the oscillator strength for excitations, a high value of which is necessary for efficient emission [29]. In the lowest S**_1_** states, the holes of BP-3-Cz are mainly concentrated in the *N*-phenylcarbazole, whereas the electrons disperse mostly on the benzophenone, which indicates an ICT transition. In S_2_ states, BP-3-Cz obviously exhibits a n-π* transition, mainly on the benzophenone. Too-large twist angles (such as 90°) between the carbazole and benzophenone produce a pure ICT (S_0_ → S_1_**)** with total spatial separation of orbitals at a higher energy level (3.44 eV), which is closer to that of dark (n, π*) (S_0_ → S_2_ 3.62 eV). Therefore, the electron transition is almost suppressed due to both the low *f* (0.0008) of S_0_ → S_1_ and proximity effect of dark S_2_ to S_1_. Weak or even quenched emission will be observed in the highly twisted conformation of BP-3-Cz. With a decrease in the torsion angle, a few holes and electrons are extended to the benzophenone and carbazole, respectively. Thus, the increased orbital overlap in the excited state facilitates the radiative transition and leads to higher *f* at low twist angles (0.5302 at 20°). Meanwhile, the energy level is decreased to 3.34 eV, which is farther away from that of dark (n, π*). Consequently, the relatively smaller torsion angle and more planar conformation help maintain stronger fluorescence. The strong fluorescence of BP-3-Cz in high-polarity solvents infers that the high polarity could induce a more planar structure and larger conjugation to achieve a more stable PICT with a decreased S_1_ energy level, which is far from that of S_2_ (n-π*) [18].

## 3. Materials and Methods

All chemicals were purchased from commercial sources and used without further purification, including 9-phenyl-9*H*-carbazol-3-ylboronic acid (98%), 4-bromobenzophenone (98%), Pd(PPh_3_)_4_ (99%), potassium carbonate (98%), anhydrous magnesium sulfate (99.5%), etc. All the other solvents are commercially available and used as received, unless otherwise claimed.

^1^H NMR and ^13^C NMR were obtained using an Avance III nuclear magnetic resonance spectrometer from Bruker, Fällanden, Switzerland, with tetramethylsilane (TMS) as the internal standard, and deuterated chloroform (CDCl_3_) as the solvent. Low-resolution mass spectrometry (MS) data were collected on a ThermoFisher ITQ1100 mass spectrometer and bombarded with an ESI source. Fourier transform infrared spectrometry (FTIR) was recorded on a Nicolet 6700 (Thermo Fisher Nicolet, Waltham, MA, USA). The ultraviolet-visible absorption spectrum (UV-Vis) was tested on a Shimadzu UV-2600 UV-visible spectrophotometer from Kyoto, Japan. The photoluminescence (PL) spectra were tested on a SENS-9000 steady-state fluorescence spectrometer from Gilden Photonics, Glasgow, UK. The PL quantum efficiencies were obtained using a C11347-11 absolute quantum efficiency meter from Japan’s Hamamatsu Company, Shizuoka, Japan. The lifetimes of the luminogens were tested on a Hitachi F7000 transient fluorescence spectrometer (Hitachi, Tokyo, Japan). Powder X-ray diffraction (XRD) was performed on an X’Pert Pro from Panaco, Amsterdam, The Netherlands, using Cu-Kα at 40 kV and 40 mA. Differential scanning calorimetry (DSC) was performed on a TA Instruments DSC2920 at a scanning rate of 10 °C min^−1^ (TA Instruments, New Castle, DE, USA). The ground state geometry was optimized using DFT, and the excited states were calculated with linear response time-dependent DFT (TDDFT) at the optimized ground state geometry. All calculations were performed with the Gaussian 16 package using the hybrid B3LYP function and the 6-311G* basis set. Grimme’s D3BJ dispersion correction was used to improve calculation accuracy. Hole–electron analysis and orbital energy level analysis were performed using the Multiwfn package. The visualization of the orbitals and hole–electron distributions were achieved using VMD software.

Synthesis of phenyl(4-(9-phenyl-9*H*-carbazol-3-yl)phenyl)methanone (BP-3-Cz): Under nitrogen atmosphere, a mixture of 9-phenyl-9*H*-carbazol-3-ylboronic acid (0.57 g, 2 mmol), 4-bromobenzophenone (0.63 g, 2.4 mmol), Pd(PPh_3_)_4_ (0.043 g, 0.04 mmol), K_2_CO_3_ (2.0 M, 3.0 mL), and toluene (50 mL)/THF (30 mL) was stirred at 90 °C for 24 h. After it was cooled to room temperature, 100 mL of CHCl_3_ was added to the mixture. The organic portion was separated and washed with brine before it was dried over anhydrous MgSO_4_. The solvent was evaporated off, and the solid residues were purified by column chromatography to afford 0.60 g of BP-3-Cz with a yield of 70.9%. ^1^H NMR (400 MHz, CDCl_3_): δ (ppm) 8.43 (s, 1H), 8.21 (d, *J* = 7.6 Hz, 1H), 7.94 (d, *J* = 8.3 Hz, 2H), 7.87 (d, *J* = 7.4 Hz, 2H), 7.84 (d, *J* = 8.5 Hz, 2H), 7.72 (d, *J* = 8.6 Hz, 1H), 7.66–7.59 (m, 5H), 7.53 (d, *J* = 7.8 Hz, 2H), 7.50 (d, *J* = 8.5 Hz, 2H), 7.45–7.42 (m, 2H), 7.33 (t, *J* = 8.0 Hz, 1H). ^13^C NMR (100 MHz, CDCl_3_): δ (ppm) 196.45, 146.10, 141.42, 140.85, 137.94, 137.43, 135.42, 132.28, 131.97, 130.89, 130.00, 129.98, 128.29, 127.69, 127.07, 126.95, 126.37, 125.44, 124.00, 123.31, 120.41, 120.28, 119.11, 110.25, 110.04. FTIR (KBr, cm^−1^) *ν* aromatic C–H 3051 (w); C=O 1650 (s); aromatic C=C 1597, 1054, 1450 (s); C–N 1282 (s); γ C–H 702 (s). MS *m*/*z*: calculated for C_31_H_21_NO, 423.2; found [M + H]^+^, 424.2.

## 4. Conclusions

In conclusion, an *N*-phenylcarbazole-substituted benzophenone derivative (BP-3-Cz) with a twisted D–A structure was synthesized. Aside from showing AIE and pressure-induced emission, BP-3-Cz exhibits an unusual PIE behavior. It has a faint fluorescence in Hex, with a PLQY of only 0.3%; while in high-polarity DMF and ACN, it has a particularly strong fluorescence, with PLQYs more than 90%. This special optical property is attributed to two causes: (1) the weak fluorescence of BP-3-Cz in low-polarity solvents and crystalline solid originates from a suppressed electronic transition in highly twisted conformation due to the close-lying S_2_ (n, π*) and S_1_ energy levels and especially low *f* of S_0_→S_1_; (2) the strong emission is released owing to the molecular conformation change to planarization in high polarity or under grinding to facilitate radiative transition (increased *f*) and enlarge the energy gap between S_2_ (n, π*) and S_1_. These findings aid in the understanding of the PIE mechanism and the design of new PIE materials.

## Figures and Tables

**Table 1:** Detailed photophysical data of BP-3-Cz in different solvents.

Solvents	Hex	Tol	THF	DCM	Ace	DMF	ACN
*ε*	1.90	2.38	7.58	8.93	20.7	37	37.5
*n*	1.375	1.494	1.407	1.424	1.359	1.427	1.344
*f*	0.0012	0.014	0.210	0.217	0.284	0.276	0.305
*λ_abs_* (nm)	331	338	338	339	331	342	337
*λ_flu_* (nm)	370	394	380	463	471	489	500
*υ_a_* (×10^4^ cm^−1^)	3.021	2.959	2.959	2.950	3.021	2.924	2.967
*υ_f_* (×10^4^ cm^−1^)	2.703	2.538	2.632	2.160	2.123	2.045	2.000
*ν_a_* − *ν_f_* (×10^3^ cm^−1^)	3.18	4.21	3.27	7.90	8.98	8.79	9.67
Φ_PL_^1^ (%)	0.3	0.7	1.2	63.9	41.3	90.6	91.4

^1^ Absolute PLQYs.

## Data Availability

All data are contained within the article.

## Sample Availability

Samples of the compounds are available from the authors.

## References

[1] Teng M.-J., Jia X.-R., Yang S., Chen X.-F., Wei Y. (2012). Reversible Tuning Luminescent Color and Emission Intensity: A Dipeptide-Based Light-emitting Material. Adv. Mater..

[2] Sun J., Lv X., Wang P., Zhang Y., Dai Y., Wu Q., Ouyang M., Zhang C. (2014). A donor–acceptor cruciform p-system: High contrast mechanochromic properties and multicolour electrochromic behavior. J. Mater. Chem. C.

[3] Li Y., Liu Y., Zhou H., Chen W., Mei J., Su J. (2017). Ratiometric Hg^2+^/Ag^+^ Probes with Orange Red-White-Blue Fluorescence Response Constructed by Integrating Vibration-Induced Emission with an Aggregation-Induced Emission Motif. Chem. Eur. J..

[4] Ma Y., Zhang Y., Kong L., Yang J. (2018). Mechanoresponsive Material of AIE-Active 1,4-Dihydropyrrolo[3,2-b]pyrrole Luminophores Bearing Tetraphenylethylene Group with Rewritable Data Storage. Molecules.

[5] Luo J., Xie Z., Lam J.W.Y., Cheng L., Chen H., Qiu C., Kwok H.S., Zhan X., Liu Y., Zhu D. (2001). Aggregation-induced emission of 1-methyl-1,2,3,4,5-pentaphenylsilole. Chem. Commun..

[6] Sun J., Chen Y., Liang Z. (2016). Electroluminochromic Materials and Devices. Adv. Funct. Mater..

[7] Luo J., Li L.Y., Song Y.L., Pei J. (2011). A Piezochromic Luminescent Complex: Mechanical Force Induced Patterning with a High Contrast Ratio. Chem. Eur. J..

[8] Sattar F., Feng Z., Zou H., Ye H., Zhang Y., You L. (2021). Dynamic covalent bond constrained ureas for multimode fluorescence switching, thermally induced emission, and chemical signaling cascades. Org. Chem. Front..

[9] Mei J., Leung N.L.C., Kwok R.T.K., Lam J.W.Y., Tang B.Z. (2015). Aggregation-Induced Emission: Together We Shine, United We Soar!. Chem. Rev..

[10] Xie P., Wang J.-Y., Huang Y.-Z., Wu X.-M., Chen Z.-N. (2022). Heteroctanuclear Au_4_Ag_4_ Cluster Complexes of 4,5-Diethynylacridin-9-One with Luminescent Mechanochromism. Molecules.

[11] Zhang Y., Sun J., Zhuang G., Ouyang M., Yu Z., Cao F., Pan G., Tang P., Zhang C., Ma Y. (2014). Heating and mechanical force-induced luminescence on–off switching of arylamine derivatives with highly distorted structures. J. Mater. Chem. C.

[12] Wang Y., Zhong Y., Wang Q., Yang X.-F., Li Z., Li H. (2016). Ratiometric Fluorescent Probe for Vicinal Dithiol-Containing Proteins in Living Cells Designed via Modulating the Intramolecular Charge Transfer–Twisted Intramolecular Charge Transfer Conversion Process. Anal. Chem..

[13] Li K., Cui J., Yang Z., Huo Y., Duan W., Gong S., Liu Z. (2018). Solvatochromism, acidochromism and aggregation-induced emission of propeller-shaped spiroborates. Dalton Trans..

[14] Sun J., Yang S., Wu J., He X., Zhang Y., Ji J., Zhang C., Liang Z. (2022). In-situ electro-polymerization of fluorescent electrochromic thin films based on charge-transfer complexes. Chem. Eng. J..

[15] Ouyang M., Zhuo C., Cao F., Pan G., Lv C., Yang S., Li C., Zhang C., Sun J., Zhang Y. (2020). Organogelator based on long alkyl chain attached excimer precursor: Two channels of TICT, highly efficient and switchable luminescence. Dye. Pigment..

[16] Ghosh R., Palit D.K. (2015). Effect of Donor–Acceptor Coupling on TICT Dynamics in the Excited States of Two Dimethylamine Substituted Chalcones. J. Phys. Chem. A.

[17] Sun J., Dai Y., Ouyang M., Zhang Y., Zhan L., Zhang C. (2015). Unique torsional cruciform p-architectures composed of donor and acceptor axes exhibiting mechanochromic and electrochromic properties. J. Mater. Chem. C.

[18] Wu H., Du L., Luo J., Wang Z., Phillips D.L., Qin A., Tang B.Z. (2022). Structural modification on tetraphenylpyrazine: From polarity enhanced emission to polarity quenching emission and its intramolecular charge transfer mechanism. J. Mater. Chem. C.

[19] Wang J., Yang Y., Jiang C., He M., Yao C., Zhang J. (2022). Ultrapure deep-blue aggregation-induced emission and thermally activated delayed fluorescence emitters for efficient OLEDs with CIE_y_ < 0.1 and low efficiency roll-offs. J. Mater. Chem. C.

[20] Yang S., Lin Y., Sun J., Li C., Zhang Y., Zhang C. (2022). Integrated electrochromic and electrofluorochromic properties from polyaniline-like polymers with triphenylacrylonitrile as side groups. Electrochim. Acta.

[21] Li W., Pan Y., Yao L., Liu H., Zhang S., Wang C., Shen F., Lu P., Yang B., Ma Y. (2014). A Hybridized Local and Charge-Transfer Excited State for Highly Efficient Fluorescent OLEDs: Molecular Design, Spectral Character, and Full Exciton Utilization. Adv. Optical Mater..

[22] Li C., Ye L., Sun J., Zhang C., Song Q., Wang K., Ouyang M. (2022). Near-infrared piezochromism of AIE-active luminophore in hybridized local and charge-transfer excited state—The effect of shortened donor-acceptor distance. Dye. Pigments.

[23] Zhou C., Han X., Liao G., Zhou C., Jin P., Guo Y., Gao H., Zhang Y., Yang S., Sun J. (2019). A Fluorescent Chemosensor with a Hybridized Local and Charge Transfer Nature and Aggregation-Induced Emission Effect for the Detection of Picric Acid. ChemistrySelect.

[24] Sun J., Liang Z. (2016). Swift Electroflfluorochromism of Donor−Acceptor Conjugated Polytriphenylamines. ACS Appl. Mater. Interfaces.

[25] Li H., Han J., Zhao H., Liu X., Luo Y., Shi Y., Liu C., Jin M., Ding D. (2019). Lighting Up the Invisible Twisted Intramolecular Charge Transfer State by High Pressure. J. Phys. Chem. Lett..

[26] Roy B., Reddy M.C., Hazra P. (2018). Developing the structure–property relationship to design solid state multi-stimuli responsive materials and their potential applications in different fields. Chem. Sci..

[27] Yu Y., Ma L., Yang X., Zhou H., Qin H., Song J., Zhou G., Wang D., Wu Z. (2018). High Efficiency Fluorescent Electroluminescence with Extremely Low Efficiency Roll-Off Generated by a Donor–Bianthracene–Acceptor Structure: Utilizing Perpendicular Twisted Intramolecular Charge Transfer Excited State. Adv. Opt. Mater..

[28] Lu T., Chen F. (2012). Multiwfn: A multifunctional wavefunction analyzer. J. Comput. Chem..

[29] Zhang Y., Qile M., Sun J., Xu M., Wang K., Cao F., Li W., Song Q., Zou B., Zhang C. (2016). Ratiometric pressure sensors based on cyano-substituted oligo(*p*-phenylene vinylene) derivatives in the hybridized local and charge-transfer excited state. J. Mater. Chem. C.

